# Volume Rendering of Angiographic Optical Coherence Tomography Angiography in Fovea Plana and Normal Foveal Pit

**DOI:** 10.3389/fneur.2021.633492

**Published:** 2021-04-27

**Authors:** Serena Fragiotta, Chiara Ciancimino, Andrea Perdicchi, Alessandro de Paula, Solmaz Abdolrahimzadeh, Gianluca Scuderi

**Affiliations:** ^1^Ophthalmology Unit, Department of Neurosciences, Mental Health, and Sensory Organs (NESMOS), St. Andrea Hospital, University of Rome “La Sapienza”, Rome, Italy; ^2^Azienda Ospedaliera San Giovanni-Addolorata, Rome, Italy

**Keywords:** fovea avascular zone, fovea plana, optical coherence tomography angiography, superficial vascular complex, deep vascular complex, microvascular 3D visualization technology

## Abstract

This paper aims to study adaptative vascular arrangements in idiopathic fovea plana with volume-rendered optical coherence tomography angiography (OCTA). A retrospective review of two cases of idiopathic fovea plana (mean age: 26.5 years) and two age-matched controls imaged with OCTA was conducted using spectral-domain OCTA (RTVue XR Avanti, Optovue, Inc., Fremont, CA) equipped with the AngioVue software. Both en face OCTA slabs and OCTA b scans were processed through Fiji software (http://fiji.sc; software version 2.0.0-rc-68/1.52e), and then extracted as image sequences for volume rendering reconstructions using the ImageVis3D volume rendering system (3.1.0 release). Eyes with idiopathic fovea plana demonstrated a regular superficial vascular plexus connecting to a single vascular monolayer representing the deeper vascular plexuses. At this location, several vertical short path connections were demonstrated, in contraposition with normal eyes where short path connections were infrequently observed. Advances in three-dimensional OCTA reconstruction increase the understanding of vascular connections and arrangement in retinal plexuses and possible anatomical variations that cannot be detected with conventional two-dimensional b scans.

## Introduction

Fovea plana is a descriptive term introduced by Marmor et al. (1) to characterize the anatomical absence of the foveal pit without functional implications and thus to introduce a clear distinction with the term foveal hypoplasia which carries a negative functional connotation. Foveal hypoplasia has been referred to as a loss of foveal depression associated with poor vision and nystagmus due to an arrest in development. The centrifugal displacement of the inner retinal cells is responsible for the foveal pit formation; it starts at 25 weeks of gestation and terminates at around 15 months of age (2, 3). Foveal hypoplasia can be observed clinically in various diseases such as ocular albinism, aniridia, and retinopathy of pre-maturity (ROP) (3–5).

Fovea plana has been described as a type of idiopathic foveal hypoplasia that is not linked to congenital abnormalities nor functional defects. It has an incidence that ranges from 1.7 to 3% in normal children (2) and is described as a bilateral condition, more commonly presenting as grade 1 or grade 2 foveal hypoplasia according to Thomas et al. (3) classification by which the lower grades correspond to a better visual prognosis. It can be diagnosed thanks to optical coherence tomography (OCT) scans, where the absence of the foveal pit, or the presence of a very shallow pit, are visualized readily.

Recently, three-dimensional rendering of high-resolution microscopic specimens and *in vivo* optical coherence tomography angiography (OCTA) helped to characterize the retinal vascular anatomy in the macular region (6, 7). This brief report aims to explore the microvascular retinal adaptations in cases of absence of the foveal pit as occurs in fovea plana. The volume rendering analysis enables us to transform two-dimensional b scans obtained through projection resolved OCTA into a three-dimensional reconstruction of the entire microvascular network and the interconnections between retinal plexuses.

## Methods

A retrospective case series of subjects with idiopathic fovea plana and age-matched controls presented at the University of Rome Sapienza-St. Andrea Hospital were examined through a volumetric rending of spectral-domain OCTA. The research followed the tenets of the Declaration of Helsinki, and all patients gave their written informed consent for publication of their cases and images.

### Subjects

Patients were included if they presented complete medical records, including complete demographics, manifest and patent refraction, best-corrected visual acuity, anterior and posterior segment examination, and OCTA examination. The diagnosis and foveal hypoplasia grading were performed by two experienced retinal specialists (S.F. and S.A.) using OCT and OCTA images.

Exclusion criteria included severe ocular media opacities and ocular co-morbidity including glaucoma, diabetic retinopathy, and systemic diseases. Vitreoretinal surgery, previous intravitreal therapy, ocular surgery within the past 6 months, poor-quality images, motion artifacts, or myopia >3 diopters were also considered as exclusion criteria.

### OCTA Imaging

All participants were imaged on spectral-domain OCT/OCTA (RTVue XR Avanti, Optovue, Inc., Fremont, CA) equipped with the AngioVue software (version 2017.1.0.151; Optovue Inc.). The device scanning speed is 70,000 A-scans per second offering an optical axial resolution of 5 μm. Signal strength index (SSI) was set to 50, and a scan quality of 9/10 was considered as an acceptable cutoff for image quality.

The OCTA was performed using a 3 × 3 mm cube (Angio Retina 3.0 mm) centered onto the fovea containing 304 × 304 A-scans (2 repeats/B scans). The software automatically estimated the percentage area occupied by vasculature, called vessel density (VD), within the central 1 mm foveal center and 0.5–1.5 mm rim of the parafoveal area including superior and inferior hemifields parafoveal zone densities and temporal, superior, nasal, and inferior zone densities (8).

The foveal center was identified as the patients presented a grade 1 foveal hypoplasia, characterized by a shallow, but still present, foveal pit and outer retinal layer and ellipsoid band extrusion. Furthermore, they showed an optimal visual acuity to fix the central light target, which was helpful in centering the 3-mm retinal volume cube.

The AngioVue software automatically identifies the retinal boundaries delimiting the superficial plexus from the inner border of the inner limiting membrane (ILM) to the inner border of the inner plexiform layer (IPL, −10 μm), and the deep plexus from the inner border of the IPL–inner nuclear layer (INL) junction (−10 μm) and the outer border of the outer plexiform layer (OPL, +10 μm). The deep plexus as segmented by the software comprises both the intermediate capillary plexus (ICP) and deep capillary plexus (DCP). If the automated segmentation resulted in being inaccurate, the retinal layers were manually segmented by a single examiner (S.F.) using the “Edit band” tool and then “Save.”

### Vascular Layer Segmentation

The vascular layer segmentation was performed manually to stratify superficial, intermediate, and deep plexus according to the previously described anatomical stratification of the retinal vascular networks (7, 9–11). The superficial vascular complex (SVC) was traced between the inner border of the ILM and the inner boundary of the IPL boundary. The ICP was segmented between the inner boundary of the IPL and the inner half of the INL. The DCP was outlined between the outer half of the INL and the outer boundary of the OPL.

### Image Processing

A volume rendering based on the OCTA en face slabs was obtained using a customized manual segmentation of the vascular plexuses performed by a single experienced retinal specialist (S.F.). The three customized OCTA slabs (i.e., superficial, intermediate, and deep plexus) were exported as single images and processed through Fiji software (http://fiji.sc; software version 2.0.0-rc-68/1.52e) (12). Once imported into the Fiji software, the images were binarized and merged (Image > Color > Merge Channels) assigning the following colorimetric scale: superficial as red, intermediate as yellow, and deep as blue.

A total of 12 en face OCTA slabs were binarized and colored with Fiji and then imported into the open-source ImageVis3D volume rendering system (3.1.0 release) (13) to create a volume rendering of the en face view of the retinal vascular plexuses. For further details, see Supplementary Figure 1.

Four hundred and eight OCTA b scans with flow overlay of the central 1-mm were adjusted for brightness and contrast and then false-colored through Adobe Photoshop (software version 20.0.5; Adobe, Inc., San Jose, California, USA) using the same colorimetric scale (superficial as red, intermediate as yellow, and deep as blue). Therefore, the image sequence was analyzed through ImageVis3D software. The imaging processing is summarized in Supplementary Figure 2.

## Results

This retrospective observational case series considered four eyes from two cases presenting idiopathic fovea plana on clinical and multimodal imaging examination (mean age of 26.5 ± 2.1 years). Clinical features of the cases examined are reported in Table 1.

**Table 1:** Clinical findings in subjects with fovea plana.

**Characteristic**	**Patient 1**	**Patient 2**
Gender	Male	Male
Age, years	25	28
Fundus pigmentation	Normal	Normal
Best corrected visual acuity	20/20	20/20
Nystagmus	Absent	Absent
FAZ	Absent	Absent
Iris color	Blue	Blue
Foveal Pit (on OCT)	Shallow	Shallow
Foveal hypoplasia	Grade 1	Grade 1
**Vessel density (whole)**		
SVC (%)	51.3	48.3
DCP (%)	57.8	52.7
**Vessel density (fovea)**		
SVC (%)	39.8	40.7
DCP (%)	61.8	54.5

*FAZ, Foveal avascular zone detected on optical coherence tomography angiography. Foveal hypoplasia grading was assessed using Thomas et al. classification (3). SVC, superficial vascular complex; DCP, deep capillary plexus*.

### Case Presentation

#### Case 1

**Figure 1:**
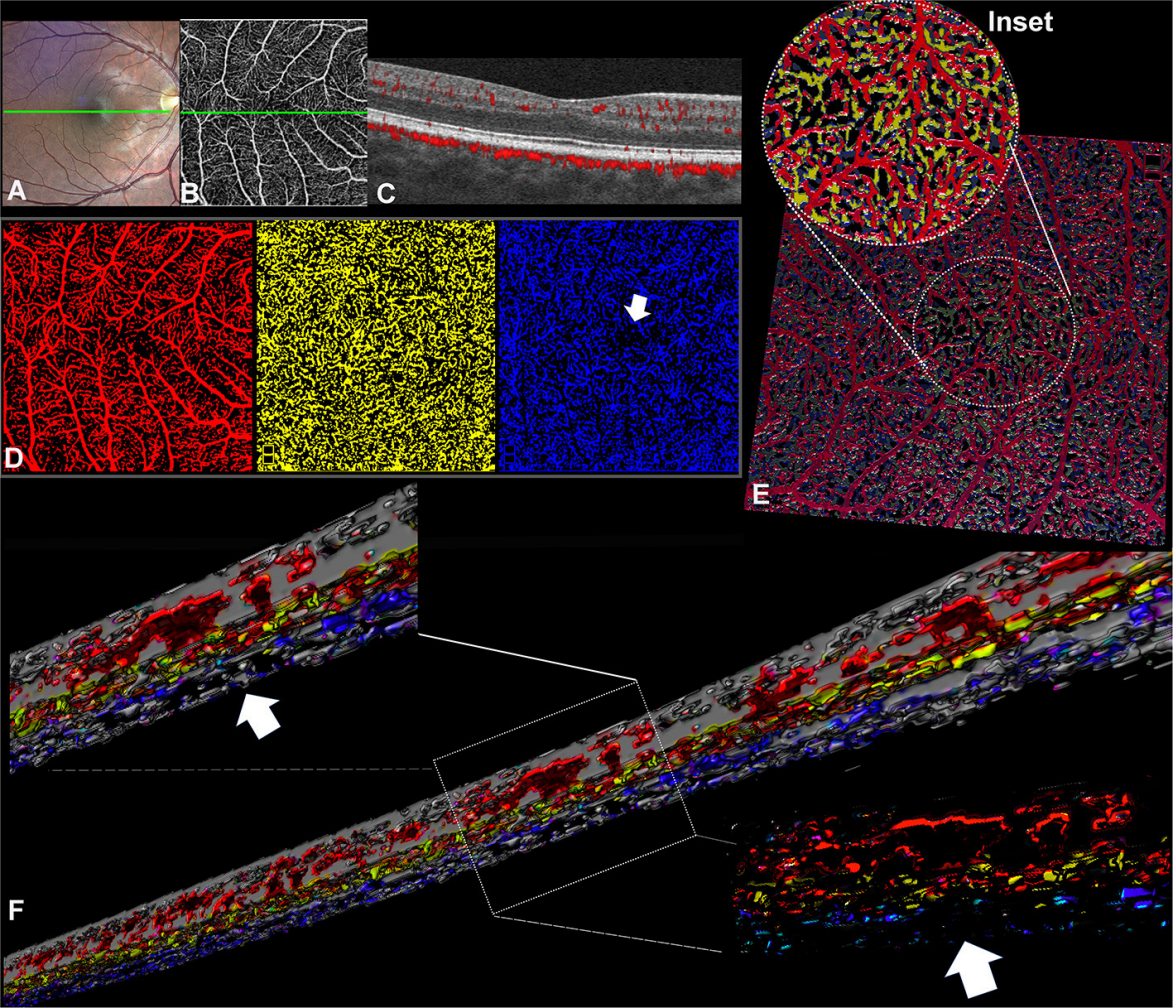
Optical coherence tomography angiography (OCTA) processing in a case of idiopathic fovea plana (Case 1). **(A)** TrueColor Confocal Fundus Imaging (Compass, Centervue, Padova, Italy). **(B)** En face OCTA (RTVue XR Avanti, Optovue, Inc., Fremont, CA) depicting the superficial vascular complex (SVC) with the retinal capillary network crossing the macular area. **(C)** The OCTA b scan obtained in correspondence of the green line confirms the fovea plana appearance. **(D)** En face OCTA slabs binarized and false-colored, the SVC (red) and ICP (yellow) show retinal capillaries occupying the macular region without a discernible avascular zone, while the DCP (blue) demonstrates a discrete central avascular area (white arrow). **(E)** Volume rendering obtained from the binarized and false-colored en face slabs allows better visualization of the spatial relation between SVC and ICP in the central macular region where the DCP is not visible. **(F)** Three-dimensional reconstruction of the OCTA b scans shows the absence of the vascular net at the level of the DCP but several interconnections between the SVC and ICP.

A 28-year-old male presented for a routine examination with no visual complaints. He was born pre-term at 33 weeks and was assisted with ventilation; his personal history was negative for ROP but presented a unilateral left preauricular cyst. He also denied family history of albinism or other retinal diseases. The best-corrected visual acuity was 20/20 with a refractive error of +1.25 diopters spherical equivalent. Upon OCT examination foveal hypoplasia was diagnosed as a grade 1 hypoplasia according to the classification of Thomas et al. (3) characterized by a very shallow foveal pit and absence of the extrusion of the plexiform layers (Figure 1C).

After segmentation and binarization of the en face OCTA slabs (Figure 1), the SVC (red) and the ICP (yellow) exhibited a continuous arrangement of retinal capillaries extending through the central macula. Instead, the DCP (blue) demonstrated a central decreased vascular network resembling a small avascular zone. The retinal capillary interconnections within the macular region can be better appreciated with the 3D en face reconstruction (Figure 1E, inset), where the SVC and ICP are colocalized with no evidence of the DCP in the center. Furthermore, the 3D reconstruction of the OCTA b scans offered further evidence of the absence of the DCP in the center despite several interconnections detectable between SVC and ICP at the same level (Figure 1F). In the parafoveal region, the vertical connections between ICP and DCP can be clearly appreciated.

#### Case 2

**Figure 2:**
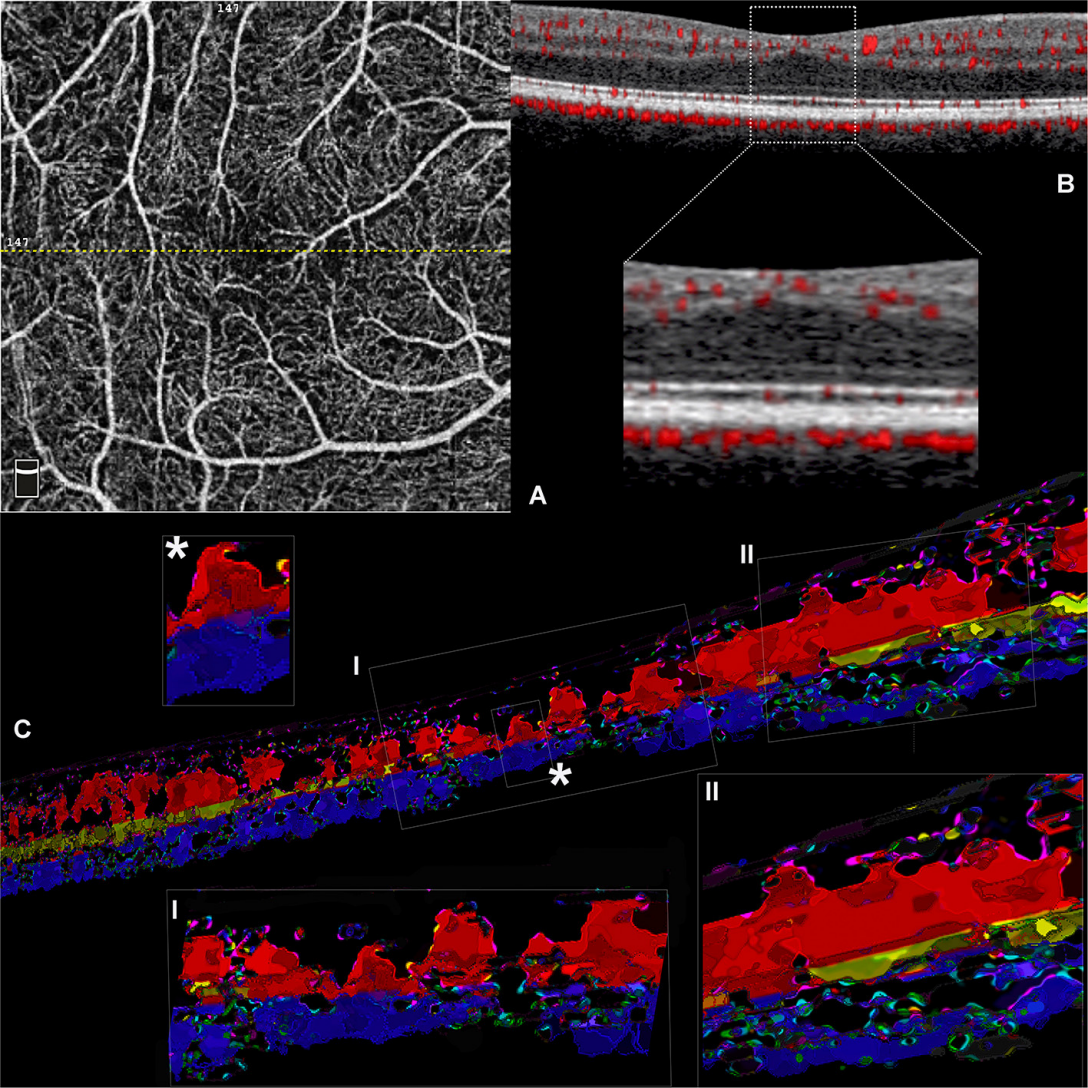
Volume rendering of optical coherence tomography angiography (OCTA) b scans obtained from a case of idiopathic foveal plana (Case 2). **(A)** En face OCTA slab representing the superficial plexus; the dotted yellow line traces the exact location of the OCTA b scan reported. **(B)** OCTA b scan demonstrating the distribution of flow signal with no detectable ICP in the central region (inset). **(C)** Three-dimensional reconstruction obtained from consecutive central OCTA b scans. Inset I with further magnification (*) shows the existence of several shortest path connections between SVC and DCP within the central 0.5 mm. However, the regular three-vascular-plexus organization is recovered at the level of parafovea, where the interconnections between SVC and ICP are well visible.

A 25-year-old male presented for routine ophthalmologic examination without visual symptoms and a negative history of albinism and any other retinal disease. The best-corrected visual acuity was 20/20 with a refractive error of +1.50 diopters spherical equivalent. His OCT findings revealed a grade 1 foveal hypoplasia, according to the classification of Thomas et al. (3) (Figure 2B). The OCTA examination confirmed the absence of a detectable foveal avascular zone (Figure 2A), and the morphological conformation of the foveal region (Figure 2B, inset) revealed the absence of a discernable ICP. In this case, the anatomical segmentation of the en face OCTA slabs was inaccurate for the irregular contour of the INL at this level. For this reason, the OCTA b scan segmentation and subsequent analysis appeared more accurate and reproducible, as shown in Figure 2.

It is worthy of note, in this case, that the ICP was not apparent, and the SVC and DCP exhibited direct vertical connections in the central macular region (Figure 2C, insets). At the parafoveal region, the ICP appeared discernible again, presenting multiple interconnections with the SVC and the DCP.

### Control Cases

Two age-matched (mean age: 27.5 years, two males) healthy subjects served as controls. The best-corrected visual acuity was 20/20 in both eyes, with normal anterior segment examination, applanation tonometry, and fundus examination. The personal and family medical history were negative for albinism.

**Figure 3:**
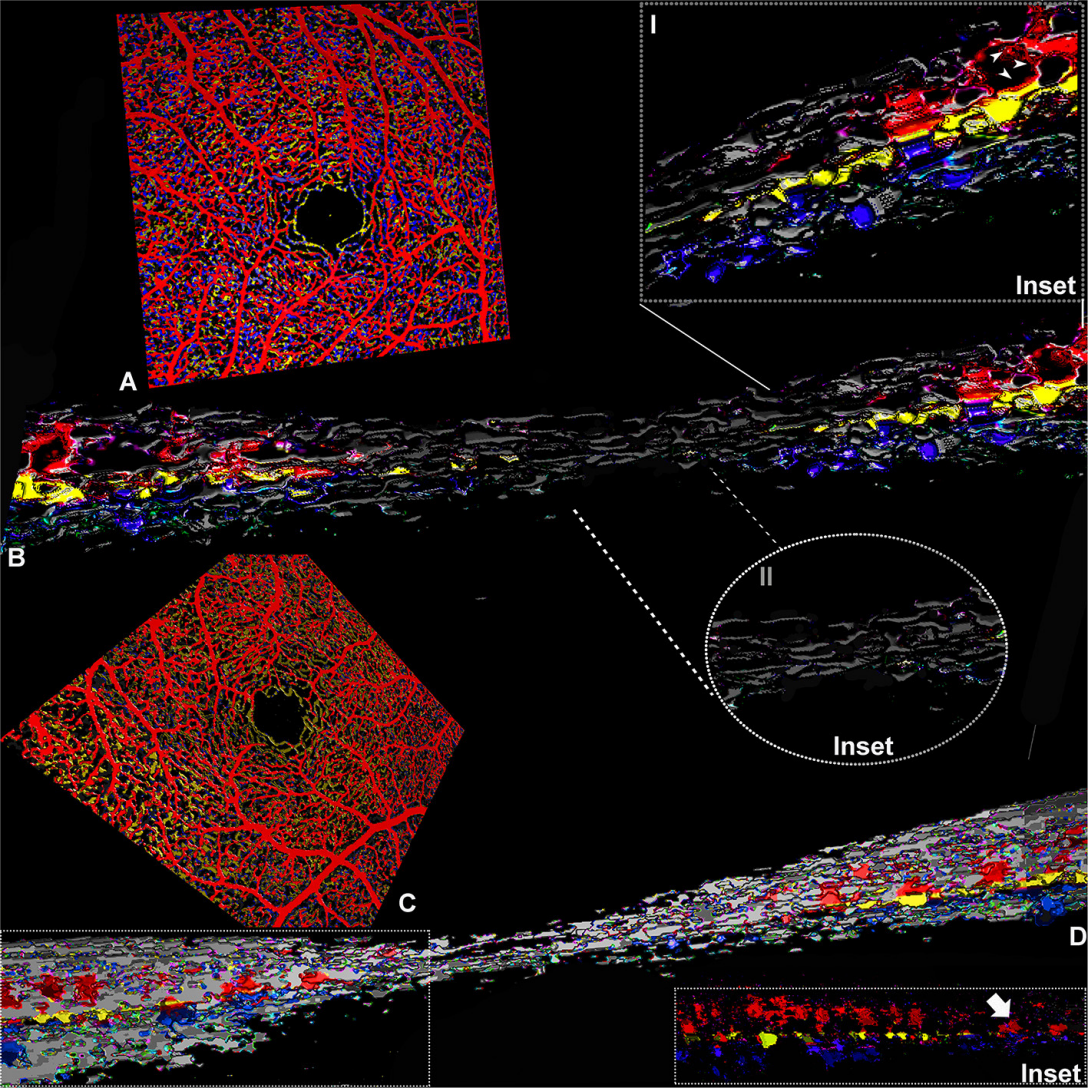
Three-dimensional evaluation of the capillary networks in healthy subjects. Two different representative cases, a 27-year-old male (Case 3) and a 28-year-old male (Case 4), are illustrated. Case 3: **(A)** Volume rendering of the en face OCTA slabs segmented as superficial (red), intermediate (yellow), and deep (blue). **(B)** Volume rendering obtained on consecutive OCTA b scans with a magnified parafoveal region (Inset I) and foveal avascular region (Inset II). Case 4: **(C)** Volume rendering of the en face OCTA slabs. **(D)** 3D reconstruction of consecutive OCTA b scans; the inset shows the shortest paths between SVC and DCP infrequently observed in normal eyes (white arrow).

On OCTA examination, the foveal avascular zone was detectable in both cases, and the first vascular branches contouring the avascular area within the central 0.5 mm were constituted by capillaries originating from ICP and DCP (Figures 3A,C). At this level, several vertical connections between ICP and DCP can be appreciated on 3D rendering of the consecutive b scans (Figures 3B,C). In the parafovea, the connection between retinal capillary plexuses was particularly abundant between the SVC and ICP, followed by connections between ICP and DCP, but on rare occasions, the SVC connected directly to the DCP (Figure 3D, inset).

## Discussion

The introduction of OCTA improved visualization and understanding of the deeper capillary plexuses, ICP and DCP, which cannot be visualized through conventional fluorescein angiography. Gattoussi and Freund (11) demonstrated the anatomical distinction between ICP and DCP vasculature by manually segmenting the retinal boundaries on OCTA b scans following previously determined histopathological findings (14). Different morphological appearances on en face OCTA further corroborated the anatomical distinction between these two vascular plexuses; the ICP presented a tortuous configuration with multiple interconnections between capillaries, while the DCP was organized into vortices where capillaries convey toward an epicenter (11, 15).

Volume rendering reconstructions substantially improved the comprehension of the macular microcirculation and interactions between the three capillary plexuses (6, 7, 16). Consistent with previous histologic and *in vivo* studies (9, 17–20), in the control cases our findings indicate that most connections intervene between the SVC and the ICP and less frequently between ICP and DCP. In normal subjects, direct connections between SVC and DCP were rarely demonstrated in the parafovea. Contrariwise, in the case of fovea plana, direct connection between SVC and DCP increase in the foveal region, with a return to the typical configuration in the parafoveal and perifoveal regions.

The common denominator of our cases is the absence of trilaminar vascular flow, which seems to be replaced by a “monolayer” of uncertain vascular phenotype representing the deeper vascular plexus. Following the previously described topographical distribution of the vascular plexuses (14), our findings suggest that blood flow in the macular region may vary according to foveal conformation but always maintaining several direct connections with the deeper vascular networks. The presence of a residual central avascular zone in the DCP demonstrable in some cases of idiopathic fovea plana has been already described by Dolz-Marco et al. (21). In our case, the high number of interconnections between SVC and ICP suggested an alternative pathway to satisfy the metabolic demand of the retinal layers occupying the foveal region.

It has been hypothesized that the absence of the avascular zone leads to merging of the SVC and DCP into a single foveal capillary layer (20). The arterial and venous blood flow have different pathways within the trilaminar network; it seems that arterial blood can reach the DCP only by passing through the ICP, while the venous outflow has been shown to occur also directly from the DCP to SVC. For this reason, direct connections between SVC and DCP have been shown rarely in the normal human fovea (7). In our study, the use of a 3D reconstruction of the capillary network demonstrated the existence of the shortest path connections between the SVC and a single deeper capillary network that, following the anatomical distribution previously described (11), can be represented by either ICP or DCP.

From a developmental perspective, the formation of foveal depression starting from the last part of gestation proceeds along with the development of foveal avascular zone (FAZ) (22). As these processes are strongly interconnected, this may explain the peculiar anatomical distribution of this developmental anomaly. It is noteworthy that, despite the incomplete development of foveal morphology, the presence of in-series interconnections may suggest their essential role in the normal macular hemodynamics.

In our series, one patient presented a pre-term history, which has been demonstrated to affect the foveal development with a reduced or absent FAZ and foveal depression, representing a distinctive signature of pre-maturity and an indicator of visual function (23–25). However, in our case, visual acuity was preserved despite the anatomical foveal shallowing and FAZ absence.

Limitations of the present study include its retrospective nature; the small case series that consists of only two cases of foveal hypoplasia, which are moreover of the same hypoplasia grade; and two healthy controls. Furthermore, the manual segmentation adopted to discriminate between ICP and DCP can lead to segmentation errors. Another limitation was represented by the qualitative assessment of the 3D imaging without quantitative measurements that can be useful to increase the repeatability of the measurements. Despite these limitations, our small series identified an alternative structural arrangement of capillary networks in response to the anatomical lack of foveal depression. From the analysis of the b scans within the central 1 mm, the microvasculature in the foveal region appeared to follow an in-series arrangement with several interconnections between SVC and the deep circulation, despite the loss of a trilaminar organization of the retinal vascular plexuses. Further studies are necessary to confirm our findings on larger series; beyond its complexity, the use of 3D reconstruction would be beneficial to fully understand the retinal microvascular network.

## Data Availability Statement

The original contributions presented in the study are included in the article/Supplementary Material, further inquiries can be directed to the corresponding author.

## Ethics Statement

The studies involving human participants were reviewed and approved by Rif. CE 5606_2020 Comitato Etico dell'Universita' Sapienza *via* di Grottarossa 1035-1039, 00189 - ROMA Tel. 06.3377.5374/5806/5662. The patients/participants provided their written informed consent to participate in this study. Written informed consent was obtained from the individual(s) for the publication of any potentially identifiable images or data included in this article.

## Author Contributions

SF and CC contributed equally to the conception and design, data acquisition, and analysis and interpretation. AdP contributed to data acquisition and elaboration. AP and SA offered an interpretation of the analysis and critical review. GS coordinated research execution and critical review of the manuscript. All authors contributed to the article and approved the submitted version.

## Conflict of Interest

The authors declare that the research was conducted in the absence of any commercial or financial relationships that could be construed as a potential conflict of interest.

## Supplementary Material

Supplementary Figure 1 The image composition describes the imaging processing for the optical coherence tomography angiography (OCTA) en face slabs. The processing is reported for each single retinal capillary plexus [i.e., superficial (SVC), intermediate (ICP), and deep (DCP)] as outlined on the right side, where the segmentation used is also shown. The OCTA slabs were binarized and colored using the color merge function on Fiji software (http://fiji.sc; software version 2.0.0-rc-68/1.52e). The images obtained were then exported as an image sequence (.tiff format) and then processed through the ImageVis3D volume rendering system (3.1.0 release).

Supplementary Figure 2 The composite illustrates the image processing conducted on optical coherence tomography angiography (OCTA) b scans. On the left side, the different steps are labeled as “original,” depicting the original OCTA b scan obtained from the subjects; “adjusted,” representing the OCTA b scan adjusted for brightness and contrast on Fiji software (http://fiji.sc; software version 2.0.0-rc-68/1.52e); “recolored,” demonstrating the different retinal plexuses colored by using a pre-set colorimetric scale, where the superficial vascular plexus (SVC) is represented as red, the intermediate as yellow and the deep as blue. The image processing was repeated for each single OCTA b scan enclosed in the central 1-mm (~101 consecutive B scans). After creating an image sequence, progressively enumerated (from top to bottom), it was imported to obtain a volume rendering into the ImageVis3D volume rendering system (3.1.0 release).
