# Supplementary figures and images for: Volume Rendering of Angiographic Optical Coherence Tomography Angiography in Fovea Plana and Normal Foveal Pit
Source: Front Neurol. 2021 Apr 27;12:633492. doi: 10.3389/fneur.2021.633492 (PMC8111301; doi:10.3389/fneur.2021.633492)

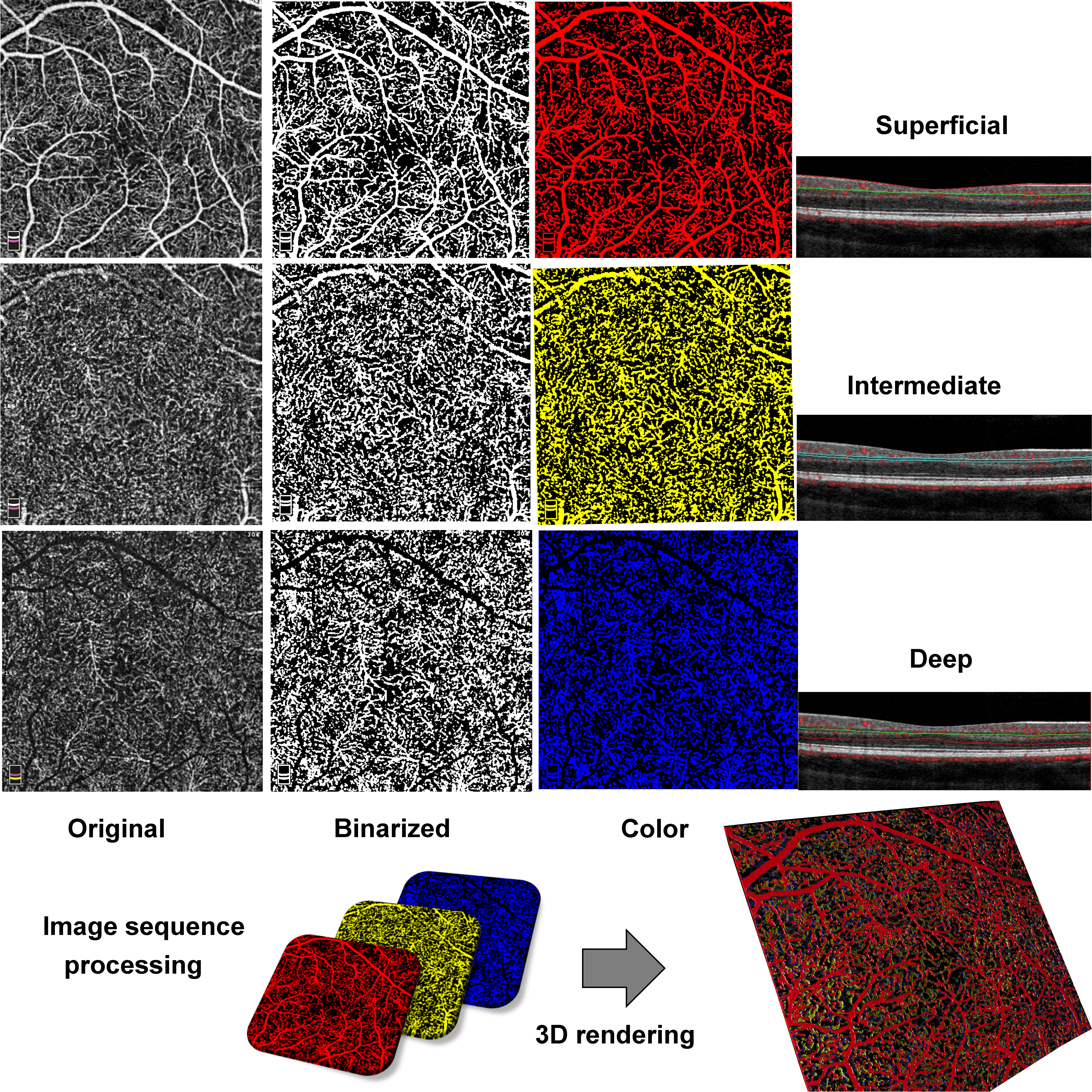

Supplement: Supplementary Figure 1 — The image composition describes the imaging processing for the optical coherence tomography angiography (OCTA) en face slabs. The processing is reported for each single retinal capillary plexus [i.e., superficial (SVC), intermediate (ICP), and deep (DCP)] as outlined on the right side, where the segmentation used is also shown. The OCTA slabs were binarized and colored using the color merge function on Fiji software (http://fiji.sc; software version 2.0.0-rc-68/1.52e). The images obtained were then exported as an image sequence (.tiff format) and then processed through the ImageVis3D volume rendering system (3.1.0 release). [file Image_1.JPEG]

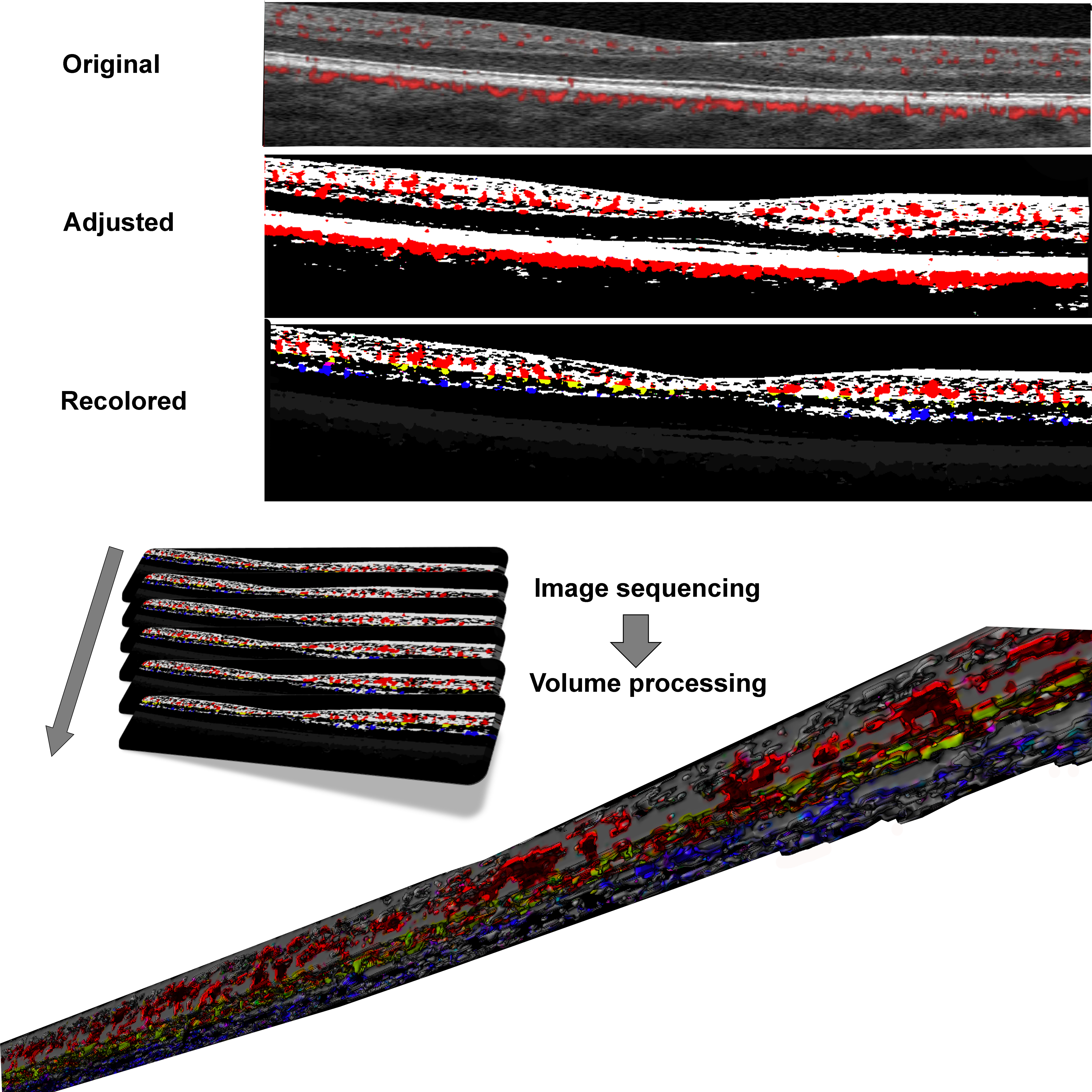

Supplement: Supplementary Figure 2 — The composite illustrates the image processing conducted on optical coherence tomography angiography (OCTA) b scans. On the left side, the different steps are labeled as “original,” depicting the original OCTA b scan obtained from the subjects; “adjusted,” representing the OCTA b scan adjusted for brightness and contrast on Fiji software (http://fiji.sc; software version 2.0.0-rc-68/1.52e); “recolored,” demonstrating the different retinal plexuses colored by using a pre-set colorimetric scale, where the superficial vascular plexus (SVC) is represented as red, the intermediate as yellow and the deep as blue. The image processing was repeated for each single OCTA b scan enclosed in the central 1-mm (~101 consecutive B scans). After creating an image sequence, progressively enumerated (from top to bottom), it was imported to obtain a volume rendering into the ImageVis3D volume rendering system (3.1.0 release). [file Image_2.JPEG]
